# A Preliminary Report on the CO2 Laser for Lumbar Fusion: Safety, Efficacy and Technical Considerations

**DOI:** 10.7759/cureus.262

**Published:** 2015-04-04

**Authors:** Alan T Villavicencio, Sigita Burneikiene, Jason M Babuska, Ewell L Nelson, Alexander Mason, Sharad Rajpal

**Affiliations:** 1 Neurosurgery, Boulder Neurosurgical Associates; 2 Neurosurgery, Justin Parker Neurological Institute

**Keywords:** co2 laser, laser-assisted spine surgeries, transforaminal lumbar fusion

## Abstract

The purpose of this study was to evaluate potential technical advantages of the CO_2_ laser technology in mini-open transforaminal lumbar interbody fusion (TLIF) surgeries and report our preliminary clinical data on the safety and clinical outcomes. There is currently no literature discussing the recently redeveloped CO_2 _laser technology application for lumbar fusion. Safety and clinical outcomes were compared between two groups: 24 patients that underwent CO_2_ laser-assisted one-level TLIF surgeries and 30 patients that underwent standard one-level TLIF surgeries without the laser.

There were no neural thermal injuries or other intraoperative laser-related complications encountered in this cohort of patients. At a mean follow-up of 17.4 months, significantly reduced lower back pain scores (P=0.013) were reported in the laser-assisted patient group compared to a standard fusion patient group. Lower extremity radicular pain intensity scores were similar in both groups. Laser-assisted TLIF surgeries showed a tendency (P = 0.07) of shorter operative times that was not statistically significant.

Based on this preliminary clinical report, the safety of the CO_2_ laser device for lumbar fusion surgeries was assessed. There were no neural thermal injuries or other intraoperative laser-related complications encountered in this cohort of patients. Further investigation of CO_2_ laser-assisted lumbar fusion procedures is warranted in order to evaluate its effect on clinical outcomes.

## Introduction

Laser-assisted spine surgeries for conditions associated with degenerative disc disease are perceived as a more effective treatment among laypersons. However, this surgical tool has received less favorable feedback from the majority of spine surgeons due to the lack of clinical data. There are currently no clinical trials that demonstrate improved clinical outcomes of any laser-assisted procedures over traditional surgical methods for this indication [1-2]. The majority of published clinical studies in the literature at this time evaluate percutaneous laser-assisted discectomies [2-4], which, like any other intradiscal therapies, make an attempt to vaporize a small amount of the nucleus pulposus, in these cases with laser energy. This theoretically results in reduced intradiscal pressure, thus decreasing pressure on the nerve roots. Although some patients with contained herniations can benefit from it, according to a systematic review performed by Gibson and Waddell, clinical outcomes following percutaneous laser-assisted microdiscectomy are worse than standard microdiscectomy [3]. The application of this procedure is restricted by very limited exposure and a reduced ability to eliminate the cause of pain, such as a free fragment disc herniation, which is inaccessible with this technique. There are currently no studies reported in the literature that describe laser-assisted lumbar fusions.

Recently redeveloped CO_2_ laser technology [5] that is based on the flexible delivery of CO_2_ laser energy (BeamPath Neuro Laser, OmniGuide, Cambridge, MA) enables no-touch vaporization of the disc material and scar tissue. Proponents of laser-assisted discectomy talk of minimizing trauma inadvertently associated with pulling and tearing when conventional instruments for discectomy are utilized. This laser may also have the ability to cauterize the nociceptive fibers within the wall of the annulus [6] and allow ablation of the tissue in the lateral recess, including potentially osteophytes and durable connective tissues. This technology could be used as a supplementary tool during discectomy or fusion surgeries by potentially simplifying the actual disc removal process.

The purpose of this study was to evaluate potential technical advantages with using this laser technique in mini-open transforaminal lumbar interbody fusion (TLIF) surgeries and report our preliminary clinical data on the safety of CO_2_ laser technology by comparing clinical outcomes of CO_2_ laser-assisted cases with those of standard TLIFs performed over the same time period.

## Materials and methods

Safety and clinical outcomes were compared between the two groups: 24 patients that underwent CO_2_ laser-assisted one-level TLIF surgeries and 30 patients that underwent standard one-level TLIF surgeries concurrently performed from November of 2008 to November of 2009. The mean follow-up was 17.4 months (range: 7 – 32). The safety of spine surgeries was assessed by monitoring all intraoperative and perioperative surgical technique-related complications. In addition, MRI scans were reviewed for possible laser-related thermal injury complications. The efficacy was evaluated by documenting and comparing postoperative lower back and leg pain (10-point Visual Analog Scale, VAS), Patient Satisfaction with Results survey scores and fusion rates.

### Surgical technique

The surgical technique for the TLIF approach has been previously described [7-8]. Some of the TLIF surgeries were performed using the Aspen Spinous Process System (Lanx, Broomfield, CO) and unilateral pedicle screws. For the laser-assisted cases, the CO_2_ BeamPath Neuro laser (OmniGuide, Cambridge, MA) was utilized for a variety of purposes that included but was not limited to opening of the disc space, vaporization of disc material, ablation of the tissue in lateral recesses, osteophytes, and calcified portions of the annulus fibrosis along with cauterizing nociceptive receptors within the wall of the annulus. Scar tissue was also removed for those cases that were reoperations. The laser fibers have a spot size of 0.40 - 0.57 mm when used in cutting mode 2 - 3 mm from the tissue. In ablative mode, which was utilized for soft tissue vaporization, this spot size expands to ~1 mm at a distance of 20 - 30 mm from the tissue, owing to beam divergence.

A series of rongeurs, curettes, or other instruments are generally used to remove disc fragments and clean the intervertebral space to remove tissues in the spinal canal that could cause secondary compression of neural anatomy and elicit pain. Use of the flexible laser fiber facilitates efficient removal of the tissue without the need for scraping and tearing of adherent connective tissue in the lateral recess and potentially helps to avoid complications, such as nerve injury or CSF leak. There is also possibly less retraction required when using a flexible laser fiber compared to the standard instruments. The laser energy vaporizes tissue including very durable connective tissue or ossified posterior longitudinal ligament. This is potentially safer and quicker than mechanical instruments currently in use.  In cases where there were any calcification and osteophyte formation at the edge of the disc space, the laser was sometimes used at a higher power setting to help ablate these. We found that the CO2 laser is not extraordinarily effective at removing bone, and there is a possibility that such high-intensity usage of the laser can cause thermal osteonecrosis.

## Results

### Patients

Selected patient demographic and surgical characteristics are presented in Table 1 below. A total of 24 laser-assisted one-level TLIF surgeries were compared to a cohort of 30 standard one-level TLIF surgeries (no laser). There were no statistically significant differences in surgical data or patient demographics with the exception of patient age. The patients in the standard TLIF group were significantly (P = 0.0002) younger with an average age of 53 compared to 66 years of age. Patients were selected for surgery based on clinical symptoms, which included intractable low back pain and radiculopathy caused by spondylosis and/or disc herniation, foraminal and/or central stenosis, and spondylolisthesis. Seven (29%) patients in the laser-assisted group and 13 (43%) patients in the standard TLIF group had previous surgeries, which included microdiscectomies and laminectomies.

**Table 1:** Laser-assisted and standard patient group comparison P values were calculated using Fisher’s exact test, except where indicated * - t-test.

	Laser-assisted (n=24)	Standard (n=30)	P-value
Age (years)	66 (32 - 91)	53 (30 - 72)	0.0002*
Sex (F/M)	15/9	17/13	1.0
Previous surgeries	7 (29%)	13 (43%)	0.59
TLIF Levels			
L2/3	3 (13%)	2 (7%)	0.65
L3/4	1 (4%)	-	0.45
L4/5	17 (71%)	22 (73%)	1.0
L5/S1	3 (12%)	6 (20%)	0.72

### Surgical parameters

The mean estimated blood loss for one-level laser-assisted and standard TLIF surgeries was almost identical – 114 mL (range, 25 - 750) and 107 mL (range: 25 - 300), respectively. The mean surgery time for one-level laser-assisted and standard TLIF surgeries was 127 min (104 - 193) and 155 min (range: 95 - 288), respectively. Laser-assisted TLIF surgeries showed a tendency (P = 0.07) of shorter operative times that was not statistically significant.

### Safety

We did not encounter any intraoperative laser-related complications. Nine out of 24 patients (36%) had postoperative MRI scans performed. This was not standard practice, and postoperative MRIs were only performed if patients had residual symptoms, which were reviewed for possible thermal, laser-related complications. We did not see any suspicious endplate or adjacent vertebrae changes that were suggestive of laser-induced changes or injuries. All complications for the laser-assisted and standard TLIF groups are presented in Table 2 below. There were no statistically significant differences between the patient groups.

**Table 2:** Complications in laser-assisted and standard TLIF procedures * - Fisher’s exact test.

	Laser-assisted (n=24)	Standard (n=30)	P-value
Hardware malposition	1 (4%)	-	1.0*
Allograft malposition	-	1 (3%)	
Vertebral body fracture	1 (4%)	-	
CSF leak	1 (4%)	2 (7%)	
Total complications	3 (12%)	3 (10%)	

### Clinical outcomes

At the mean follow-up of 17.4 months, significantly reduced lower back pain scores (P = 0.037) were reported in the laser-assisted patient group compared to the standard fusion patient group. The lower extremity radicular pain intensity scores were similar in both groups (Table 3). The Patient Satisfaction with Results survey did not reveal any statistically significant differences between the groups (Table 4). Two (8.3%) patients in the laser-assisted fusion group had pseudoarthrosis. Similarly, two (6.7%) patients in the standard TLIF group had incomplete fusions and two (6.7%) had pseudoarthrosis. This difference was not statistically significant (P = 0.68, Fisher’s exact test).

**Table 3:** Lower back and leg pain scores Preop: preoperative, Postop: postoperative, VAS: Visual Analog Scale (P values were calculated using t-test.)

	Laser-assisted (n=24)	Standard (n=30)	P-value
Preop VAS back	6.7 (0 – 10)	7.8 (0 – 10)	0.16
Postop VAS back	1.7 (0 – 8)	3.5 (0 – 8)	0.013
Preop VAS leg	7.0 (0 – 10)	6.8 (0 – 10)	0.84
Postop VAS leg	2.1 (0 – 10)	2.4 (0 – 9)	0.71

**Table 4:** Patient satisfaction with results survey P values were calculated using Fisher’s exact test

	Patient Responses	Laser-assisted (n=24)	Standard (n=30)	P-value
How satisfied are you with the treatment you received?	Dissatisfied (1/5)	1 (4.2%)	0	P = 0.39
	Somewhat dissatisfied (2/5)	0	0	
	Don’t know (3/5)	3 (12.5%)	2 (6.6%)	
	Somewhat satisfied (4/5)	4 (16.6%)	5 (16.7%)	
	Very satisfied (5/5)	16 (66.7%)	23 (76.7%)	
	Score (range)	4.4 (1 -5)	4.7 (3 - 5)	
How is your pain or conditions that you had surgery for now compared to before surgery?	Much worse (1/5)	0	2 (5.9%)	P = 1.0
	Worse (2/5)	1 (4.2%)	2 (5.9%)	
	Same (3/5)	4 (16.6%)	2 (5.9%)	
	Better (4/5)	6 (25%)	12 (41.2%)	
	Much better (5/5)	13 (54.2%)	12 (41.2%)	
	Score (range)	4.3 (2 -5)	4.0(1 – 5)	
Would you have surgery again for the same condition?	Definitely no (1/5)	1 (4.2%)	0	P = 1.0
	Probably no (2/5)	1 (4.2%)	2 (5.9%)	
	Don’t now (3/5)	5 (20.8%)	7 (23.5%)	
	Probably yes (4/5)	2 (8.3%)	14 (47.1%)	
	Definitely yes (5/5)	15 (62.5%)	7 (23.5%)	
	Score (range)	4.2 (1 - 5)	3.9 (2 - 5)	

## Discussion

### Safety

There were no neural thermal injuries or other intraoperative laser-related complications encountered in the group of patients that underwent laser-assisted TLIFs that we are aware of. Although this is a preliminary report with a limited follow-up time, the authors feel that the minimum seven-month follow-up after surgery in these patients is a sufficient time for potential laser-induced symptoms to become apparent. Previously reported complications utilizing a percutaneous approach include major artery perforation [9], nerve root injury [10], infectious discitis [4], and thermal endplate and vertebral body necrosis [11].

A series of 13 patients that underwent salvage operations after failed percutaneous laser-assisted microdiscectomies were reported [11]. Laser-induced intradisc CT defects were observed in three patients, and high-intensity MRI changes were observed in the adjacent vertebrae in eight out of 10 patients. These changes were identified as a separation between the endplate and the vertebral body due to thermal injury during laser-assisted procedures performed with Ho: YAG laser. The disc tissue resected during salvage operations contained carbonized lesions. All patients that developed postoperative neurological deficits had large compressive herniated discs with adhesions to the nerve root. The authors hypothesized that such thermal injuries to the endplates containing vascular beds may promote disc degeneration or induce disc instability.

The CO_2 _laser that was utilized in this report is unique in that it has a very high absorption in water and minimal lateral thermal spread in tissue (with a high water content). This theoretically reduces the risk of thermal injury to the adjacent structures [12] and makes this technique potentially safer than other laser-assisted spine surgical technologies. In 1997, Nerubay, et al. [13] reported six patients with CT and MRI changes in the endplates but no correlation with clinical outcomes in a series of 50 patients that underwent percutaneous CO_2_ laser-assisted nucleolysis using a total of 960J laser energy. An additional safety feature has since been added to the redesigned Omni Guide CO_2_ laser system utilized in the present cases – a cooling gas, helium, that is released at the fiber’s tip. This added an additional protection against thermal injuries. Another issue related to percutaneous laser discectomy is that clinical outcomes and complications are heavily dependent on perfect needle position. This is not the case with mini-open techniques, which is what we describe in the current paper.

### Effectiveness and clinical outcomes

Several previously reported potential advantages of laser-assisted discectomies include reduced bleeding and swelling [9], hemostasis and sterilization with cutting [12], and the ability to reduce perineural scar tissue formation [14]. There was also an experimental study that investigated the CO_2_ laser effect on epidural fibrosis where a defocused laser was used to vaporize the fibrotic tissues on the dura matter and nerve roots after laminectomy on guinea pigs [15]. A significant decrease in the amount of fibrosis, fibroblastic activity, and collagen mass formation without affecting normal nerve tissues was demonstrated. All these properties could theoretically contribute to improved clinical outcomes; however, it remains unclear whether the same mechanisms would have preventable measures in clinical settings.

Our preliminary results demonstrated significantly reduced lower back pain scores (P = 0.0213) in the laser-assisted TLIF patient group compared to the standard fusion patient group. The mechanism of this improvement is not clear, and these findings would have to be replicated in future prospective randomized clinical studies. The standardized clinical outcome measurement tools were not employed in this study, and we can only make preliminary conclusions about the efficacy of laser-assisted fusion surgeries. We have considered that a placebo effect could have resulted in this symptomatic improvement. However, the fact that the study was not randomized and patient satisfaction survey scores were similar in both groups reduces such a possibility. This improvement could potentially be related to the ability of the laser to cauterize the nociceptive fibers and thoroughly clean the intervertebral space.

This study also suggested a marginally reduced operative time for laser-assisted compared to standard lumbar fusion surgeries. It is possible that the CO_2_ laser is more efficient at removing disc material than standard instruments and thus requires less time to perform a discectomy. Further confirmation of the OR time-saving benefits is also needed. The laser fibers that we utilized are disposable and costly. An economic analysis that includes longer-term clinical outcomes is therefore also necessary before this technology becomes widely available. 

## Conclusions

Based on this preliminary non-randomized clinical data, the safety of the CO_2_ laser device for lumbar fusion surgeries was assessed. There were no neural thermal injuries or other intraoperative laser-related complications encountered in this cohort of patients. Further investigation of CO_2_ laser-assisted lumbar fusion procedures is warranted in order to evaluate its effect on clinical outcomes.
